# Are Animals As Irrational As Humans?

**DOI:** 10.1371/journal.pbio.0020434

**Published:** 2004-11-23

Animals in the wild are constantly confronted with decisions: Where to nest? Who to mate? Where's the best forage? To explore the mechanisms underlying such decisions, animal behavior studies often incorporate concepts from economic theory. Mainstream models of choice in both economics and biology predict that preferences will be rational, or consistent across contexts, as a result of being motivated by self interest or, in the case of animals, reproductive success. Yet many studies report that when making decisions people often take shortcuts, using cognitive heuristics that may lead to economically irrational decisions, with similar claims now showing up in animal behavior studies.

In a new study, Cynthia Schuck-Paim, Lorena Pompilio, and Alex Kacelnik ask whether studies applying economic rationality to animal behavior are controlling for potentially confounding effects inherent in such approaches. The authors suggest that observed “breaches of rationality” may stem from differences in the physiological state of animals “unwittingly imposed” by experimental design rather than from real irrational decisions.

Choice studies typically offer subjects a range of choices that include clearly superior and inferior alternatives. While humans can simply hear about the various alternatives and their respective properties, animals must be trained to learn about the different choices. This difference is far from trivial, Schuck-Paim et al. argue, and could well require different interpretations of results in animal and human studies. In fact, economic theory states that optimal choices depend on both the properties of the option and the chooser's state. Training animals to learn of different choices typically involves giving them food rewards, which means that an animal's energetic state—that is, hungry versus sated—will change over a day of training. A bird that's eaten an ounce of birdseed is more likely to opt for an “irrational” option—say, a choice that dispenses little food—than one that's hungry.

To examine this theoretical constraint under experimental conditions, Schuck-Paim et al. trained European starlings to choose between two rich food sources (called focal options) and one of two poorer “decoys” in different contexts. One of the focal options offered more food while the other offered a shorter delay between pecking a key and receiving the food, but their amount/delay ratios were equal. The decoys were considered less preferable because their ratio of amount to delay was lower than that of the focal options. But the decoys could potentially confound the results because repeated training to each decoy could sate a bird's appetite to different degrees: although amount/delay was equal among the decoys, their long term energetic consequences differed.[Fig pbio-0020434-g001]

**Figure pbio-0020434-g001:**
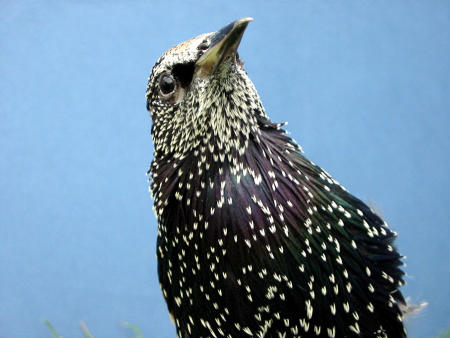
European starlings make rational decisions

The authors tested for preference between the focal options under three experimental conditions: altering the birds' food intake/energetic state with no decoys; changing the decoy and not controlling for its corresponding energetic contributions; and changing the decoy but controlling for its energetic consequence (by supplemental feeding).

Schuck-Paim and collaborators show that the birds' preferences between the focal options differed significantly between treatments, in apparent breach of economic rationality; the preference for the larger reward option over the shorter delay option was much stronger when the trial involved lower accumulated intake than when the accumulated intake was high. Introducing the decoys resulted in an “irrational” preference only when the decoys were allowed to have an effect on food intake, suggesting that the choice resulted from the birds' energetic state rather than from cognitive mechanisms of choice similar to those used to explain irrationality in human subjects.

The authors offer an evolutionary and mechanistic explanation for why animal preference might be governed by energetic state, including the possibility that animals are less motivated to focus exclusively on the richest option when they are well fed. But they are careful to disabuse the notion that “state-dependence accounts for all reported inconsistencies in animal choice” or that animals do not employ cognitive mechanisms of choice similar to those of humans. Altogether, Schuck-Paim and co-authors argue, these results warn that studies appropriating ideas from other disciplines can introduce confounding effects. And that researchers would do well to carefully examine the underlying causes of observed animal behaviors when testing ideas formulated in a nonbiological framework.

